# Inter-Individual Variability in Xenobiotic-Metabolizing Enzymes: Implications for Human Aging and Longevity

**DOI:** 10.3390/genes10050403

**Published:** 2019-05-27

**Authors:** Paolina Crocco, Alberto Montesanto, Serena Dato, Silvana Geracitano, Francesca Iannone, Giuseppe Passarino, Giuseppina Rose

**Affiliations:** Department of Biology, Ecology and Earth Sciences, University of Calabria, 87036 Rende, Italy; crocco.paola@gmail.com (P.C.); alberto.montesanto@unical.it (A.M.); serena.dato@unical.it (S.D.); silvana.geracitano@unical.it (S.G.); francescaiannonebio@gmail.com (F.I.)

**Keywords:** aging, longevity, survival, SNP, polymorphism, xenobiotic-metabolizing enzymes, xenobiotics

## Abstract

Xenobiotic-metabolizing enzymes (XME) mediate the body’s response to potentially harmful compounds of exogenous/endogenous origin to which individuals are exposed during their lifetime. Aging adversely affects such responses, making the elderly more susceptible to toxics. Of note, XME genetic variability was found to impact the ability to cope with xenobiotics and, consequently, disease predisposition. We hypothesized that the variability of these genes influencing the interaction with the exposome could affect the individual chance of becoming long-lived. We tested this hypothesis by screening a cohort of 1112 individuals aged 20–108 years for 35 variants in 23 XME genes. Four variants in different genes (*CYP2B6*/rs3745274-G/T, *CYP3A5*/rs776746-G/A, *COMT*/rs4680-G/A and *ABCC2*/rs2273697-G/A) differently impacted the longevity phenotype. In particular, the highest impact was observed in the age group 65–89 years, known to have the highest incidence of age-related diseases. In fact, genetic variability of these genes we found to account for 7.7% of the chance to survive beyond the age of 89 years. Results presented herein confirm that XME genes, by mediating the dynamic and the complex gene–environment interactions, can affect the possibility to reach advanced ages, pointing to them as novel genes for future studies on genetic determinants for age-related traits.

## 1. Introduction

Aging is a complex phenotype responding to a plethora of drivers in which genetic, behavioral, and environmental factors interact with each other. This can be conceptualized in terms of exposome—that is, the totality of exposures to which an individual is subjected throughout a lifetime and how those exposures affect health [1].

The exposome basically includes a wide variety of toxic or potentially harmful compounds of exogenous (environmental pollutants, dietary compounds, drugs) or endogenous (metabolic by-products such as those resulting from inflammation or lipid peroxidation, oxidative stress, infections, gut flora) origin and related biological responses during the life course [2].

The individual ability to properly cope with xenobiotic stress can influence susceptibility to diseases and, thus, the quality and the rate of aging, phenotypes that certainly result from the cumulative experiences over lifespan. Additionally, in all the different theories proposed to explain the aging process, a common denominator remains the progressive decline of the capacity to deal with environmental stressors to which the human body is constantly exposed.

In this scenario, a crucial role can be played by the coordinated activity of cellular mechanisms evolved for reducing the toxicity of endogenous and xenobiotic compounds to which humans are exposed. These mechanisms comprehend a broad range of reactions of detoxification that make harmful compounds less toxic, more hydrophilic, and easier to be excreted. The main effectors of these mechanisms are a large number of enzymes and transporters, collectively referred to as xenobiotic-metabolizing enzymes (XMEs) or drug metabolizing enzymes (DMEs). This process occurs in three phases. Phase I enzymes, such as cytochrome P450s (CYPs), carboxylesterases, and flavin monooxygenases, add reactive groups to the toxin; in phase II, glutathione S-transferases (GST), UDP-glucuronosyltransferases (UGT), catechol-*O*-methyltransferases (COMT), and *N*-acetyltransferases (NAT) conjugate water-soluble groups onto the molecule; in phase III, ATP-binding cassette (ABC) transporter proteins facilitate the export of the conjugate out of cells as well as the import and the efflux of a broad range of substrates [3].

With aging, there is a decline in the ability to mount a robust response to xenobiotic insults. This is somewhat attributed to the age-related reduction in liver mass, which can result in reduced metabolism rates and in the decreased kidney and liver blood flows, which can result in reduced excretion and elimination of xenobiotic and its metabolites [4]. In addition, a reduction in the activity of phase I and II enzymes and the consequent fall in biotransformation capacity have been reported by several authors in both old animals and humans [5,6,7]. As aging is characterized by an increased prevalence of chronic conditions that require the use of multiple medications, these changes have particular relevance from a clinical point of view, affecting drug effectiveness and toxicity [8]. Moreover, transcriptional profiling has revealed the up-regulation of xenobiotic-metabolizing genes in long-lived mutants across diverse model organisms [9,10,11], suggesting that the individual ability to modulate xenobiotic responses may either lead to increased risk of diseases and death or favor longevity.

It is also known that the activity of XME proteins is affected by the variability of the corresponding genes, whose polymorphisms can account for the inter-individual variability in both xenobiotic response/toxicity and disease predisposition. In this regard, significant associations of alleles in these genes (especially in phase II genes) with many forms of cancer [12,13] or coronary heart disease [14] were found. Moreover, in testing a sample of individuals of different ages, Ketelslegers et al. [15] found that the prevalence of risk alleles in XME genes decreases with age, suggesting that individuals carrying a higher number of risk alleles show a higher risk of morbidity and mortality for chronic diseases.

Based on all the above, we reasoned that genetic variants of XME genes might affect the chance to live a long life. In order to test this hypothesis, we screened a set of 35 SNPs in 23 XME genes and their association with aging and survival in a cohort of 1112 individuals aged 20–108 years, performing both case-control and prospective cohort analyses.

## 2. Materials and Methods

### 2.1. Study Population

The initial dataset included 1112 unrelated individuals (497 men and 615 women) whose ages ranged from 20 to 108 years. All subjects were born in Calabria (Southern Italy), and their Calabrian ancestry was ascertained up to the third generation. Samples were collected within the framework of several and appropriate recruitment campaigns carried out for monitoring the quality of aging in the whole of Calabria, as previously reported [16]. In brief, younger subjects were recruited from students and staff of the University of Calabria; elderly subjects were from people visiting thermal baths, the Academy of the Elderly, or contacted through general physicians. Very old subjects were selected through the population registers and then contacted and invited to join the study. Old and very old subjects underwent a multidimensional geriatric assessment with the aim of collecting clinical history, anthropometric measures, cognitive functioning, functional activity, and physical performance.

White blood cells (WBC) from blood buffy coats were used as sources of DNA, while plasma/sera were used for routine laboratory analyses.

For the analyses, the sample was divided in three specific age classes based on two age thresholds, 65 and 89 years, after which a significant negative change in the slope of the survival curve of the Italian population occurs [17]. Thus, subjects were classified as younger adults (age class S1, 20–64 years; *n* = 330), elderly (age class S2, 65–89 years; *n* = 433), and very old subjects (age class S3, ≥90 years; *n* = 349).

For subjects of the 65- to 89-year-old group, vital status was traced after a mean follow-up time of approximately 10 years through the population registers of the municipalities where the respondents lived.

### 2.2. Ethic Statement

The study was approved by the Ethical committee of the University of Calabria (Rende, Italy, on 9 September 2004). All the subjects provided written informed consent in accordance with institutional requirements and the Declaration of Helsinki principles.

### 2.3. Cognitive and Physical Assessments

Cognitive status was assessed by age- and education-adjusted Mini Mental State Examination (MMSE) [18]. The score ranges from 0 to 30, and a score of 23 points or less is usually considered to indicate cognitive impairment. Hand grip (HG) strength was evaluated by using a handheld dynamometer (SMEDLEY’s dynamometer TTM) while the subject was sitting with the arm close to the body. Three consecutive measurements were performed with the stronger hand, and the maximum value was used for data analysis. The performance of activities of daily living (ADL) (bathing, dressing, toileting, transfer from bed to chair, and feeding) was assessed using a modification of the Katz Index [19]. Scores were dichotomized as 1 if the subject was able to perform every activity and as 0 otherwise. Depressive symptoms were assessed using the 15-item Geriatric Depression Scale (GDS) [20]. Subjects with GDS scores greater than or equal to 5 were considered to be affected by depressive symptom.

### 2.4. SNPs Selection and Primer Design for iPLEX TM Assay

A panel of 35 candidate polymorphisms was selected based on known functional consequence (exonic, regulatory regions) or prior associations with disease risk, pathology, or drug response. SNPs were chosen from 23 genes involved in detoxification related pathways of xenobiotic substances. Supplementary Table S1 reports the complete list of assayed SNPs and their basic features. In summary, we selected 6 SNPs in 5 genes of phase I, 11 SNPs in 7 genes of phase II, and 13 SNPs in 7 genes of phase III. Four non-canonical XME genes (indicated as others in Table S1) and relative polymorphisms (4 SNPs) were selected because they have been deeply studied in relation to drug response, and thus likely affect the risk or the clinical evolution of several diseases. All SNPs have a reported minor allele frequency of >0.05 in Europeans. For each polymorphism, PCR and extension primers were designed using Sequenom MassARRAY Assay Designer 3.0 software (Sequenom, San Diego, CA, USA), resulting in a 22 plex and a 13 plex.

### 2.5. Sequenom Mass Spectrometry Genotyping

First, 2 µL of genomic DNA (5 ng/uL) were PCR-amplified in a 5 µL reaction containing 0.8 µL HPLC grade water, 0.5 µL of 10 × PCR buffer with 20 mM MgCl_2_, 0.4 µL of 25 mM MgCl_2_, 0.1 µL of 25 mM dNTP mix, 1 µL of 0.5 μM primer mix, and 0.2 µL Sequenom PCR enzyme. PCR conditions were: an initial cycle at 94 °C for 2 min, 45 cycles at 95 °C for 30 s, 56 °C for 30 s, 72 °C for 60 s, and a final step at 72 °C for 5 min.

Unincorporated dNTPs in the amplification products were dephosphorylated by adding 2 µL of the shrimp alkaline phosphatase (SAP, Sequenom) mix consisting of 1.53 µL of HPLC grade water, 0.17 µL of SAP buffer, and 0.3 µL (0.5 U) of SAP enzyme (Sequenom). Each reaction was incubated at 37 °C for 40 min, and SAP was then heat inactivated at 85 °C for 5 min.

Following SAP treatment, a single base pair extension reaction was performed using Sequenom’s iPLEX Gold chemistry, where 2 µL of the iPLEX reaction mix was added to the samples. The reaction mix consisted of 0.62 µL of HPLC grade water, 0.2 µL of iPlex buffer, 0. 2 µL of iPlex terminator mix, 0.94 µL of primer mix, and 0.04 µL of iPlex enzyme. Thermal cycling conditions included an initial cycle at 94 °C for 30 s; 40 cycles at 94 °C for 5 s, [52 °C for 5 s and 80 °C for 5 s (repeat 5 times per cycle)]; and a final step at 72 °C for 3 min. The samples were then resin treated and spotted on a SpectroCHIP using the MassARRAY nanodispenser (Sequenom) and analyzed using the MassARRAY Compact System matrix-assisted laser desorption/ionization-time-of-flight mass spectrometer (MALDI-TOF) (Sequenom). Genotypes were assigned in real time using the MassARRAY SpectroTYPER RT v3.4 software (Sequenom) based on the mass peaks present. All results were manually inspected using the MassARRAY TyperAnalyzer v3.3 software (Sequenom).

### 2.6. Quality Control

After genotyping, samples were subjected to a battery of quality control (QC) tests. At sample level, subjects with a proportion of missing genotypes higher than 10% were dropped from the analysis. At SNP level, SNPs were excluded if they had a significant deviation from Hardy–Weinberg equilibrium (HWE, *p* < 0.05) in the younger subgroups, a missing frequency (MiF) higher than 20%, and a minor allele frequency (MAF) lower than 1%. See Table S1 for details.

### 2.7. Statistical Analysis

For each SNP, allele and genotype frequencies were estimated by gene counting from the observed genotypes. HWE was tested by Fisher’s exact test. Logistic regression models were used to evaluate the effect of genotypes (independent variables) on the probability of belonging to different age groups (dependent variable). Differences between age groups were tested by comparing two of them at once. Genetic data were coded with respect to a dominant, a recessive, and an additive model of inheritance. Then, for each SNP, the most likely genetic model was estimated on the basis of minimum level of statistical significance (Wald test *p*-value). In such models, sex was used as a covariate. To capture sex-dependent effects of the analyzed genetic variants, an additional interaction term was also included. Finally, to test whether combinations of SNPs might better differentiate between the different age groups, a multivariate model including the associated SNPs was also fitted. The Nagelkerke index was then used to compare the obtained models.

Linear and logistic regression models were applied to estimate the impact of genetic variability on parameters of cognitive (MMSE, GDS) and physical (HG, ADL) performance, including age, gender, and height as covariates. Continuous and categorical variables were compared by using the independent samples *t*-test and the Χ^2^ test as appropriate. For evaluating if the effect of the polymorphisms on longevity phenotype also affected the survival patterns of the different genotypes, we performed a longitudinal study after 10 years from the baseline visit. Univariate survival analysis was carried out by the Kaplan–Meier approach, and survival curves were compared by log-rank test. Subjects were considered as censored if they were alive after the follow-up time, and this time was used as censoring data in the survival analyses. Moreover, hazard ratios (HR) and 95% CI were estimated by Cox proportional hazard models using age and gender as confounder variables.

Because this was a hypothesis driven study, a level of significance *p*-value = 0.05 was considered for each association test without Bonferroni post hoc correction for multiple comparisons.

Statistical analyses were performed using SNPassoc and surv packages of R (http://www.R-project.org/).

## 3. Results

After quality control checks, there were genotyping data on 27 SNPs in a cohort of 981 individuals aged 20–108 years. Demographic characteristics for the study cohort according to age groups defined in the Materials and Methods section are presented in Table 1.

Four SNPs demonstrated a nominally significant association (*p*-value < 0.05) in at least one comparison (see Table 2).

**Figure 1:**
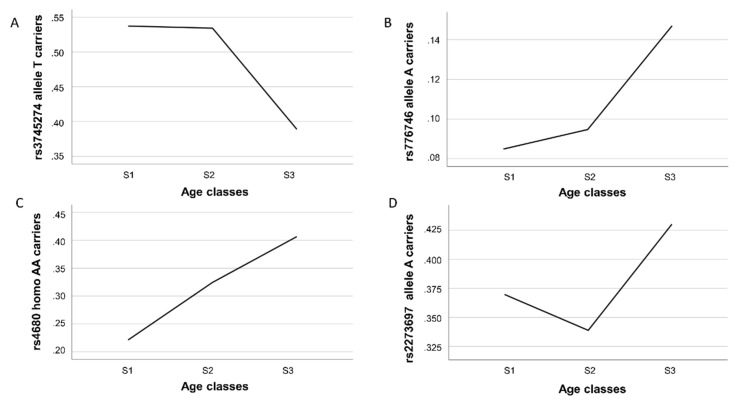
Gene frequencies across the three age classes S1 (20–64 years), S2 (65–89 years), and S3 (90–108 years) of: (**A**) T allele carriers of rs3745274 in *CYP2B6*; (**B**) A allele carriers of rs776746 in *CYP3A5*; (**C**) AA carriers of rs4680 in *COMT*; (**D**) A allele carriers of rs2273697 in *ABCC2*.

Two of them (rs3745274-G/T and rs776746-G/A) belonged to genes for phase I enzymes (*CYP2B6* and *CYP3A5*, respectively), one (rs4680-G/A) was within the phase II *COMT* gene, and one (rs2273697-G/A) was within the phase III *ABCC2* gene. The best-fitting genetic model for three of them was dominant, while rs4680-G/A best fit a recessive genetic model. As Figure 1 shows, these variants had different gene frequency trajectories over the three examined age intervals.

We found a significant decrease in the proportion of carriers of the *CYP2B6* rs3745274-T allele in the oldest sample (S3 group) with respect to the youngest S1 and S2 [odds ratio (OR) = 0.547 CI 95% 0.373–0.803, *p*-value = 0.002 for comparison 2; OR = 0.563 CI 95% 0.395–0.803, *p*-value = 0.005 for comparison 3), consistent with a detrimental effect of this allele on longevity (Table 2 and Figure 1A). An opposite effect was observed for rs776746, being carriers of the A allele significantly overrepresented in the S3 group as compared to S1 (OR = 1.97 CI 95% 1.08–3.56; *p*-value = 0.022) and S2 group. In this last case, however, only a trend toward significance was detected (OR = 1.66 CI 95% 0.99–2.79; *p*-value = 0.054) (Table 2 and Figure 1B). For both SNPs, we did not find differences between age groups in comparison 1, indicating that carrying of the above alleles confers a disadvantageous or an advantageous effect on lifespan only in the last part of life. A different trajectory was observed for the *COMT* rs4680 variant (Figure 1C). Indeed, in all the comparisons, we found that the proportion of homozygous AA individuals was always significantly higher in the oldest than in the younger subjects (OR = 1.67 CI 95% 1.10–2.54, *p*-value = 0.016 for comparison 1; OR = 2.43 CI 95% 1.58–3.73, *p*-value <0.001 for comparison 2; OR = 1.47CI 95% 1.10–2.15, *p*-value = 0.046 for comparison 3), consistent with a linear trend towards a positive effect of the rs4680-AA genotype on longevity. As for the rs2273697 variant in *ABCC2*, a significantly higher prevalence of carriers of the minor allele A in subjects belonging to the S3 group in comparison to the younger age group S2 (comparison 3) (OR = 1.459 CI 95% 1.038–2.051; *p*-value = 0.030) was observed (Figure 1D), thus indicating a beneficial impact of this allele for reaching longevity. No statistically significant evidence for genotype-by-sex interactions was observed.

Next, to evaluate the overall effect of the multivariate model on the total phenotypic variance, we estimated Nagelkerke indexes for comparisons 1 and 3; we found that the variance explained by the combined genetic data was 0.7% in comparison 1 and 7.7% in comparison 3. Finally, we evaluated the combined effect of the variability of genes listed in Table 2 at different ages. As Table S2 shows, we detected an approximately similar effect size to that seen in univariate analysis, suggesting an independent effect of each SNP. In comparison 3, three out of four SNPs included in the genetic profile remained substantially associated with the phenotype and significantly associated with the age-group membership (rs3745274-G/T, rs776746-G/A, rs4680-G/A), thus significantly discriminating very long lived (90+) from younger elderly (65–89 years old) subjects.

Since the weight of genetic factors increases starting from 65 years of age, we investigated the association of the above variants with biomarkers of age-associated changes in physical (HG and ADL) and cognitive (MMSE and GDS) abilities. A significant association was found between *COMT* rs4680 and ADL performance (*p*-value = 0.03) with subjects homozygous for the allele A showing significantly lower probability to be disabled than those carrying at least one G allele (60.7% of AA among the non-disabled vs. 39.3% of AA among the disabled). In addition, we found that the same variant significantly influenced the GDS performance in females. Subjects with the AA genotype were more represented among non-depressed than depressed individuals (78.9% vs. 51.6%; *p*-value = 0.017). No other significant association between these SNPs and geriatric parameters was observed.

**Figure 2:**
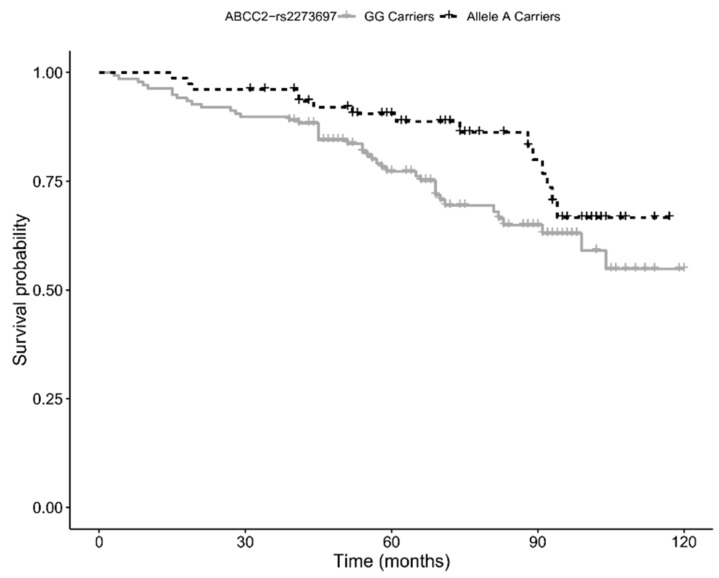
Kaplan–Meier survival functions relative to carriers of the minor allele A (black) vs. non carriers (gray) of the *ABCC2* variant rs2273697. Time is expressed in months, where zero is considered the time of recruitment, and each individual is followed up for survival status until death.

Finally, by using 10-year follow-up survival data, we assessed whether the variants we found associated with the longevity phenotype also influenced the survival of the elderly cohort (age 65–89 years). Kaplan–Meier survival analysis showed a trend for a positive association with survival for carriers of the minor allele (A) of rs2273697 in *ABCC2* (*p*-value = 0.054; see Figure 2), a result consistent with the positive effect on longevity. However, the association did not hold significance when multivariate Cox proportional hazard regression analysis was performed (HR = 0.63, 95% CI: 0.35–1.16; *p*-value = 0.143).

## 4. Discussion

In this study, we show that genetic variants of genes related to xenobiotic metabolism, such as those of phases I-III, have an influence on the chance of reaching old and very old ages beyond 100 years. Among the 27 genetic variants analyzed, four (rs3745274, rs776746, rs4680, and rs2273697) have shown to exert significantly different and age-specific effects on longevity, with changes of gene frequencies following either linear or non-linear trajectories. In particular, except for rs4680, which showed a significant linear change across the age classes, we observed major frequency changes in the other SNPs in passing from 65–89 to 90–108 age-range. In fact, the genetic variability of these genes showed to account for 7.7% of the chance to survive beyond the age of 89 years. This figure is quite important if we take into account that genetics is believed to account for 25% of the individual chance to be long-lived.

Age-specific gene effects have already been reported in literature for genetic variants in other genes thought to shape the dynamic and the complex gene–environment interactions, which profoundly change during human lifespan [21]. Genes encoding for xenobiotic metabolizing enzymes surely have these characteristics.

Completely opposite effects on longevity were observed for rs3745274-T (negative effect) and rs776746-A carriers (positive effect) located respectively in *CYP2B6* and *CYP3A5* genes that function in biotransformation reactions (phase I). Substrates for both isoenzymes include not only clinically used drugs but also a large number of environmental toxic and carcinogenic chemicals (pollutants, pesticides), as well as endogenous compounds such as steroid hormones and fatty acids [22,23].

The CYP2B6 protein makes up roughly 2–6% of total liver CYP content [24]. There is a remarkable inter-individual variability in its expression partly due to transcriptional regulation and genetic variations [22]. The rs3745274-G/T, herein found to affect the likelihood of becoming long-lived, is a missense variant (Gly516His) in exon 4. A study by Hofmann et al. [25] reported evidence that the T allele is responsible for aberrant splicing, resulting in a shorter variant lacking exon 4, 5, and 6 and decreased expression and enzymatic activity. Notably, relevant studies have established a link between this variant and a higher risk of multiple cancers [26,27,28]. Therefore, it is likely that individuals carrying the T allele, because of a lower detoxifying capability, have a higher susceptibility to cancer compared to non-carriers and thus a decreased probability of reaching advanced ages.

Emerging evidence also suggests that the rs776746-G/A variant in intron 3 of the *CYP3A5* gene may affect the individual’s risk of cancer development. The presence of the G allele creates a cryptic splice site that determines a truncated non-functional protein. Therefore, subjects carrying the GG genotype are considered to be CYP3A5 non-expressors [29]. Very interestingly, the frequency of the rs776746-G allele varies markedly across ethnic groups, ranging from about 18% in Africans to 94% in Europeans (data from 1000 genome) and is significantly correlated with population distance from the equator [30], thus suggesting considerable interaction between genotype and environment. Notably, current literature supports a relationship between the G allele and cancer risk [31,32], suggesting that a protective effect of the expressor A-allele may allow individuals to better cope with dangerous compounds potentially promoting cancer development. Consistently, we found that elderly subjects carrying the rs776746-A have a higher likelihood of becoming long-lived than non-carrying ones. Interestingly, for both rs3745274 and rs776746, changes in gene frequencies start to occur in the middle-aged group (S2), i.e., the one characterized by a higher incidence of cancer and other age-related diseases.

Moreover, the polymorphism rs2273697-G/A appeared to modulate the probability of obtaining longevity by mainly acting in the 65–89 years age group. This is a missense variant (Val417Ile) in exon 10 of the *ABCC2* gene, encoding for the multidrug resistance-associated protein 2 (MRP2), a member of the ABC transporter superfamily. This phase III protein, which is expressed in hepatocytes, renal proximal tubular cells, and enterocytes, is involved in the removal of many toxic chemicals, nutraceuticals, drugs, and their conjugates, as well as endogenous compounds (e.g., bilirubin-glucuronides) [33]. A recent finding by Wei and colleagues provided direct evidence that the rs2273697-A allele increases the ATPase and the efflux activity of the MRP2 protein [34]. Our survival analysis showed that the presence of the A allele decreases the risk of death in subjects aged 65–89 years, and that carriers of this allele are more represented in the 90+ cohort. Based on the above evidence, it seems plausible that rs2273697-A promotes increased survival at old age through an enhanced MRP2 efflux capacity, which likely results in a better protection against cytotoxic effects of toxic compounds.

Finally, we observed a significant linear increase in frequency for the *COMT* rs4680-AA genotype across the different age groups, suggesting a positive effect on survival at both old and very old ages. The rs4680 polymorphism lies in exon 4 and determines either a valine (Val; G allele) or a methionine (Met; A allele) at amino acid 158. This SNP has been reported to significantly affect enzyme activity; for instance, the Val allele has a four-fold higher enzyme activity than the Met allele [35]. COMT is an important phase II enzyme, which, by methylation, inactivates biologically active catechols (i.e., catecholamines, catecholestrogens) and toxic catechol-based molecules [36]. In recent years, COMT has become intensively studied, largely due to its role in regulation of the dopamine level in the brain. In Caucasians, the Met allele is reported to be associated with better cognitive function [37], while Val carriers tend to have a greater likelihood of becoming depressed [38]. It is well known that depression as well as cognition impairment represent major risk factors for disability, often associated with worse health outcomes and increased risk of death, especially in later life [39]. All of the above is in line with our data showing a positive effect on longevity of the A (Met) allele, moreover, confirmed by the association of the same allele with a lower depressive status and a better physical performance in elderly subjects.

On the whole, the different trends in XME gene frequencies we observed in population age once again highlight the complexity in gene–longevity associations. In fact, we found two associated SNPs behaving as pro-longevity variants (rs776746-A and rs4680-AA), one as a killing variant (rs3745274-T), while the rs2273697-A allele showed a U-like frequency curve that was higher at younger ages, decreased in early old, and then increased in exceptionally old. Such a behavior is typical of a buffered variant, in accordance with the buffering mechanism in aging hypothesis suggested by Bergman [40], which states that a deleterious variant can be neutralized by the protective effect of pro-longevity genes.

## 5. Conclusions

The present study—the first to our knowledge to investigate the association between SNPs in XME genes and human longevity—found that their variability conditions the chance to reach very old age by affecting survival in an age-specific way. This is a novel finding considering that the variability of XME genes has been extensively investigated in relation to drug metabolism and response to treatment. However, drugs are only a limited kind of substrate that XME proteins can metabolize; their detoxification function might rather modulate the dynamic and the complex biological response to the exposome, thus representing a potential determinant of longevity. We are aware that the study is not conclusive and deserves future investigations. Because the genetic variability of all the XME genes herein analyzed show population specificity, it could be very interesting to test the association with longevity in other ethnic groups. Moreover, a longer follow up time and the knowledge of specific causes of death could further support our conclusions, allowing us to better understand the specific pathological phenotype affected by the variants analyzed in this study. Nevertheless, the associations here reported may contribute to our understanding of the genetic determinants of human longevity, supporting future studies in the role of xenobiotic metabolism in quality of aging and extreme survival.

## Figures and Tables

**Table 1:** Demographic characteristics for the analyzed cohort according to age group membership.

	Age Class S1	Age Class S2	Age Class S3
*n*	287	379	315
Male%	43.6	49.1	35.9
Age Range (years) (mean, SE)	20–64 42.7 (0.89)	65–89 73.5 (0.31)	90–108 97.8 (0.75)
ADL (% Disabled)	-	43.0	69.5
GDS (% Depressed)	-	32.3	26.2
HG (Kg; mean, SE)	-	22.2 (0.62)	13.1 (0.43)
MMSE < 23 (%)	-	9.5	66.5

ADL, activity daily living; GDS, Geriatric Depression Scale; HG, hand grip; MMSE, Mini Mental State Examination; SE, standard error.

**Table 2:** Multinomial logistic analysis for univariate genetic associations.

	* Comparison 1 (Age Class 2 vs. Age Class 1)			Comparison 2 (Age Class 3 vs. Age Class 1)		Comparison 3 (Age Class 3 vs. Age Class 2)	
		65–89 Years vs. <65 Years		90+ vs. <65 Years		90+ vs. 65–89 Years	
	Gene	OR (95% CI)	*p*-Value	OR (95% CI)	*p*-Value	OR (95% CI)	*p*-Value
rs3745274-G/T	*CYP2B6*	0.97 (0.67–1.40)	0.88	0.54 (0.37–0.80)	0.002	0.56 (0.39–0.80)	0.005
rs776746-G/A	*CYP3A5*	1.20 (0.65–2.22)	0.57	1.97 (1.08–3.56)	0.022	1.66 (0.99–2.79)	0.054
rs4680-G/A	*COMT*	1.67 (1.10–2.54)	0.016	2.43 (1.58–3.73)	<0.001	1.47 (1.10–2.15)	0.046
rs2273697-G/A	*ABCC2*	0.87 (0.61–1.23)	0.44	1.26 (0.89–1.79)	0.18	1.45 (1.03–2.05)	0.030

* In each comparison, the youngest group was considered as the reference category. For both comparisons 1 and 2 (both using the youngest group as reference category), odd ratios (ORs) were obtained directly from the equations included in the models; for comparison 3 (90+ years vs. <65 years), ORs were obtained by difference of equations included in the models.

## Supplementary Materials

The following are available online at https://www.mdpi.com/2073-4425/10/5/403/s1, Table S1: List of genes and SNPs considered in the present study. Table S2. Multinomial logistic analysis for multivariate genetic associations.
