# Supplementary material for: Inter-Individual Variability in Xenobiotic-Metabolizing Enzymes: Implications for Human Aging and Longevity
Source: Genes (Basel). 2019 May 27;10(5):403. doi: 10.3390/genes10050403 (PMC6562959; doi:10.3390/genes10050403)
Supplement: Supplementary file 1 [file genes-10-00403-s001.pdf]

Table S1. List of genes and SNPs considered in the present study.

XME	Gene	SNP	Functional Consequence	AA change
**Phase I**	*CYP2A6*	rs28399433 - A/C	2KB Upstream Variant	
	CYP2B6	rs3745274 - G/T	Missense Variant	Gln172His
	CYP2E1	rs2070673 - T/A	2KB Upstream Variant	
	CYP3A5	rs776746 - C/T	Intron /Splice Acceptor Variant	
	CYP2C19	rs4244285 - G/A	Synonymous variant	Pro227Pro
		rs12248560 - C/T	2KB Upstream Variant	
**Phase II**	*COMT*	rs165599 - A/G	3 Prime UTR Variant	
		rs4680 - G/A	Missense Variant	Val158Met
	GSTP1	rs1695 - A/G	Missense Variant	Ile105Val
	NAT2	rs1801280 - T/C	Missense Variant	Ile114Thr
		rs1799930 - G/A	Missense Variant	Arg197Gln
		rs1208 - A/G	Missense Variant	Arg268Lys
	UGT1A1	rs4124874 - T/G	Intron Variant	
	UGT2B7	rs7662029 - G/A	Intron Variant	
		rs7668258 - C/T	Intron Variant	
	UGT1A6	rs2070959 - A/G	Missense Variant	Thr181Ala
	UGT1A10	rs6759892 - T/G	Missense Variant	Ser158Ala
**Phase III**	*ABCB1*	rs2032582 - A/C	Missense Variant	Ser893Thr
		rs1128503 - G/A	Synonymous variant	Gly412Gly
	ABCC2	rs2273697 - G/A	Missense Variant	Val417Ile
		rs3740066 - C/T	Synonymous variant	Ile1324Ile
	ABCG2	rs2231142 - G/T	Missense Variant	Gln141Lys
	SLC15A2	rs2257212 - C/T	Missense Variant	Leu350Phe
		rs1143671 - C/T	Missense Variant	Pro409Ser
		rs1143672 - G/A	Missense Variant	Arg509Lys
	SLC22A2	rs316019 - C/A	Missense Variant	Ala270Ser
		rs316019 - C/A	Missense Variant	Ser270Ala
	SLCO1B1	rs4149056 - T/C	Missense Variant	Val174Ala
	SLCO1B3	rs4149117 - G/T	Missense Variant	Ser112Ala
		rs7311358 - A/G	Missense Variant	Met233Ile
**Others***	*DPYD*	rs1801265 - A/G	Missense Variant	Cys29Arg
	ITGB3	rs5918 - T/C	Missense Variant	Leu59Pro
	PGTS1	rs5788 - C/A	Synonymous Variant	Gly213Gly
		rs10306114 - A/G	5 Prime UTR Variant	
	PTGS2	rs20417 - C/G	2KB Upstream Variant	

Underlined SNP were excluded from the analysis after quality control.

* These genes and relative polymorphisms were selected because deeply studied in relation to drug response, so likely affecting the risk or the clinical evolution of several diseases.

Table S2. Multinomial logistic analysis for multivariate genetic associations.

		*Comparison 1 (Age class 2 *vs* Age class 1) 65-89 years vs <65 years		Comparison 2 (Age class 3 *vs* Age class 1) 90+ vs <65 years		Comparison 3 (Age class 3 *vs* Age class 2) 90+ vs 65-89 years	
	Gene	OR (95% CI)	P-value	OR (95% CI)	P-value	OR (95% CI)	P-value
rs3745274-G/T	*CYP2B6*	1.09 (0.70-1.67)	0.702	0.62 (0.39-0.99)	0.045	0.54 (0.34-0.84)	0.006
rs776746-G/A	*CYP3A5*	1.03 (0.46-2.26)	0.946	1.86 (0.88-3.91)	0.101	1.93 (0.93-4.00)	0.075
rs4680-G/A	*COMT*	1.39 (0.84-2.32)	0.197	2.56 (1.56-4.21)	<0.001	1.93 (1.20-3.10)	0.007
rs2273697-G/A	*ABCC2*	0.97 (0.62-1.53)	0.914	1.28 (0.80-2.03)	0.30	1.30 (0.83-2.05)	0.253

*In each comparison the youngest group was considered as the reference category.

CI = Confidence interval. For both the comparisons 1 and 2 (both using the youngest group as reference category), Odd ratios (ORs) were obtained directly from the equations included in the models; for the comparison 3 (90+ years vs <65 years), ORs were obtained by difference of equations included in the models.
